# Comparison of Chinese and international birth weight standards in predicting early childhood growth outcomes: a retrospective cohort study

**DOI:** 10.3389/fped.2026.1810686

**Published:** 2026-06-08

**Authors:** Ting Zhang, Zhenyu Zhang, Mai Gao, Ziyun Li, Li Zhang, Huijuan Liu, Yan Li

**Affiliations:** 1 Shandong Provincial Maternal and Child Health Care Hospital Affiliated to Qingdao University, Jinan, Shandong, China; 2 School of Clinical Medicine, Shandong Second Medical University, Weifang, Shandong, China

**Keywords:** birth weight, growth standards, possible risk of overweight, predictive validity, wasting

## Abstract

**Background:**

Birth weight serves as a key predictor for long-term health trajectories. Currently, both international and Chinese standards are used to evaluate birth weight. This study aims to compare the Chinese Newborn Growth Standards (CNGS) and the INTERGROWTH-21st (IG-21) standards in terms of birth weight classification and their predictive performance for the wasting and possible risk of overweight during infancy and toddlerhood.

**Methods:**

This retrospective cohort study included 1,149 full-term singleton infants with longitudinal growth data recorded at 1, 2, and 3 years of age. Birth weight was categorized according to both the CNGS and IG-21 standards. Wasting and possible risk of overweight during infancy and toddlerhood were assessed according to World Health Organization (WHO) standards. Agreement between the two classification systems was evaluated using the weighted Kappa statistic. The predictive performance of each standard for growth outcomes was compared using modified Poisson regression models.

**Results:**

The CNGS and IG-21 standards showed high agreement (Kappa = 0.856). In terms of predictive performance, based on the CNGS, small for gestational age (SGA) was significantly associated with wasting at 1 and 2 years, and large for gestational age (LGA) was significantly associated with possible risk of overweight at 2 years. In contrast, based on the IG-21 standards, only SGA showed a significant association with wasting across all follow-up ages. Overall, the CNGS demonstrated higher specificity in predicting wasting and possible risk of overweight in infants and toddlers.

**Conclusion:**

Although the CNGS and IG-21 standards showed high agreement, the CNGS demonstrated superior predictive validity for growth outcomes during infancy and toddlerhood, particularly for possible risk of overweight.

## 1 Introduction

Newborns are classified as Small for Gestational Age (SGA), Appropriate for Gestational Age (AGA), or Large for Gestational Age (LGA) based on birth weight relative to gestational age (1). This classification serves not only as a primary indicator of intrauterine nutritional status but also as a crucial baseline for predicting long-term health trajectories. Deviations in birth weight—encompassing both SGA and LGA—are strongly associated with neonatal complications such as asphyxia, hypoglycemia, and respiratory distress (2–5). Furthermore, they exert profound and lasting impacts on growth and development during infancy and toddlerhood. These effects manifest across multiple domains, including physical growth (6), neuropsychological development (7–11), and metabolic (12–14) and immune function, with potential consequences extending into adulthood.

To establish a global system for neonatal growth assessment, the International Fetal and Newborn Growth Consortium for the 21st Century (INTERGROWTH-21st) published neonatal growth standards (IG-21 standards) in 2014, based on multi-national, multi-center prospective data. This project, covering populations across 8 cities in 8 countries, established international standards with broad applicability that have been widely adopted globally (15). However, while these standards offer a high degree of standardization and comparability, fetal growth is significantly influenced by ethnic and genetic backgrounds, geographical environments, nutritional patterns, and socioeconomic conditions. Therefore, constructing localized standards that accurately reflect the growth characteristics of specific population is crucial for achieving precision pediatric care and effective intervention.

The National Health Commission of China released the latest Chinese Newborn Growth Standards (CNGS) in 2022. These nationally representative standards were developed from a cross-sectional survey conducted between June 2015 and November 2018 in 13 cities. The study included 24,375 newborns with gestational ages ranging from 24 to 42 weeks. Strict exclusion criteria were applied to remove maternal or neonatal factors that could influence intrauterine growth, such as severe maternal complications, smoking, alcohol consumption, major congenital malformations, and multiple pregnancies. The CNGS provides percentiles (P3, P10, P25, P50, P75, P90, P97) for birth weight, length, and head circumference, stratified by sex and gestational age (16, 17). This offers a scientific and reliable localized basis for assessing birth weight in Chinese newborns and establishes a foundation for further research into the association between early-life growth trajectories and long-term health.

The core distinction between international and localized standards stems from the heterogeneity of the source populations. The IG-21 standard aims to establish an “ideal” international growth reference by enrolling healthy, well-nourished, low-risk pregnant women; thus, its results represent fetal growth potential under optimal conditions. In contrast, the CNGS serves as a descriptive standard, intended to reflect the actual distribution of birth weights in the contemporary Chinese newborn population. This fundamental difference suggests that applying the international standard to specific populations (such as in China) may systematically underestimate or overestimate the true proportion of SGA or LGA, thereby compromising its predictive validity for subsequent health risks. Consequently, in clinical practice and public health decision-making, the choice of standard is not merely a technical matter but a critical issue concerning screening efficiency, identification of high-risk individuals, and the effectiveness of resource allocation.

Given the close association between inappropriate birth weight and childhood health outcomes, as well as the current coexistence of international and domestic diagnostic criteria, this retrospective study aims to utilize longitudinal data from a mother-child birth cohort to systematically compare the discrepancies in birth weight classification between the CNGS and the IG-21 standards. Furthermore, it seeks to evaluate and compare their respective predictive performance for growth outcomes—specifically wasting and possible risk of overweight—during infancy and toddlerhood. The findings will help clarify whether the CNGS criteria offer greater applicability and superiority over the IG-21 for the early identification of growth deviations in Chinese infants and toddlers.

## 2 Methods

Data were derived from the Mother-Child Birth Cohort of the Shandong Provincial Maternal and Child Health Care Hospital. The ethical approval was granted by Shandong Provincial Maternal and Child Health Care Hospital Affiliated to Qingdao University (No.2025-040). Written informed consent was obtained from the parents or legal guardians of all participants.

### 2.1 Study subjects

The study screened infants and toddlers born between January 2020 and August 2021 who received regular follow-up care at the Department of Child Health Care, Shandong Maternal and Child Health Hospital. Inclusion Criteria: (1) Gestational age at birth ≥ 37 weeks and < 42 weeks; (2) Singleton pregnancy; (3) Completion of physical measurements and assessments at 1, 2, and 3 years of age. Exclusion Criteria: (1) Presence of genetic or metabolic diseases affecting growth and development; (2) Missing birth information. Based on the inclusion and exclusion criteria, a total of 1,149 infants and toddlers were enrolled in the study.

### 2.2 Data collection

Neonatal characteristics including sex (categorical), gestational age (continuous, measured in weeks), birth weight (continuous, measured in grams), and mode of delivery (categorical) were measured and recorded in the electronic medical record system by trained midwives. Maternal characteristics including maternal age at delivery (continuous, measured in years), parity (categorical), threatened abortion (dichotomous), gestational infection (dichotomous), gestational diabetes mellitus (dichotomous), gestational hypertension (dichotomous), and gestational thyroid dysfunction (dichotomous), were recorded by obstetricians. Anthropometric data and feeding information, including 1-month exclusive breastfeeding status (dichotomous), were collected during follow-up and entered into the system by child health nurses.

### 2.3 Children anthropometry

Length (cm) and weight (kg) were measured in the supine position using an integrated electronic scale (KANGWA WS-RTG-1G, Suzhou, China). The device has a length measurement range of 30–105 cm (measure to the nearest 1 mm) and a weight range of 0–60 kg (measure to the nearest 50 g). Measurements were performed independently by two healthcare professionals, and the mean value was recorded in the electronic medical record system. The maximum allowable difference between the two measurements was set at 0.7 cm for length and 0.1 kg for weight.

### 2.4 Definitions and classification

Birth weight was evaluated based on the “Growth Standards for Newborns by Gestational Age” (National Health Commission of China, 2022) and the 2014 IG-21 standards. Infants were classified into three categories: SGA (< 10th percentile), AGA (10th–90th percentile), and LGA (> 90th percentile) (1). Term infants were defined as those with a gestational age between 37^+0^ and 41^+6^ weeks (18). Low birth weight (LBW) was defined as < 2,500 g, and macrosomia as ≥ 4,000 g. Wasting and possible risk of overweight in infants and toddlers were assessed using the WHO BMI-for-age Z-score (BAZ) standards. Specifically, wasting was defined as BAZ < −2, and possible risk of overweight as BAZ > 1 (19). In this study, we utilized BAZ consistently across all ages for uniformity and direct comparability with older age groups. We also assessed the agreement between weight-for-length/height and BAZ classifications of nutritional status, which showed good agreement (Kappa = 0.853, *P* < 0.001) (Supplementary Table 1). Advanced maternal age was defined as delivery at ≥ 35 years of age (20).

### 2.5 Statistical analysis

All statistical analyses were conducted using IBM SPSS Statistics (version 27.0) and R (version 4.5.1). Comparisons of categorical variables were performed using Pearson's Chi-squared test. The consistency between the CNGS and the IG-21 birth weight classification standards was assessed using the weighted Kappa coefficient. To further evaluate the predictive performance of birth weight classifications for infant and toddler growth outcomes, sensitivity, specificity, positive predictive value (PPV), and negative predictive value (NPV) were calculated. For covariates with missing data, we employed complete-case analysis in regression models, including only participants with full data for all variables relevant to each specific model. This approach was chosen to minimize potential bias from imputation methods, particularly given the relatively low proportion (7.7%) of missing data for covariates.

To investigate the association between birth weight classification and growth outcomes, modified Poisson regression models with robust error variances were employed to estimate relative risks (RR) and their 95% confidence intervals (CI). Based on extensive evidence from existing literature and clinical relevance, the following potential confounding factors were pre-identified as fixed covariates and obligatorily included in all regression models: infant sex, maternal age at delivery (21, 22), parity (23), mode of delivery (24), threatened abortion (25), gestational infection (26), gestational diabetes mellitus (27), gestational hypertension (28), gestational thyroid dysfunction (29), and exclusive breastfeeding (30) status at one month postpartum. The selection of these variables was based on their widely established clinical and biological importance, as they are known to influence both birth weight classification and subsequent childhood growth outcomes. This strategy of obligatory inclusion, rather than stepwise selection, aimed to ensure that all known and clinically significant confounding factors were effectively controlled, thereby minimizing residual confounding and enhancing the internal validity of the estimated RR. A two-sided *P*-value < 0.05 was considered statistically significant.

## 3 Results

### 3.1 Characteristics of the participants

The study included 1,149 children (647 boys and 502 girls) and their mothers. The mean (SD) maternal age was 30.1 (3.4) years, and the majority of deliveries were vaginal (64.4%). The mean (SD) birth weight was 3,366.7 (393.6) g, and the mean (SD) gestational age was 39.5 (1.0) weeks. The prevalence of low birth weight was 0.9%, and the prevalence of macrosomia was 5.6%. The prevalence of wasting at ages 1, 2, and 3 years was 1.5%, 6.7%, and 3.7%, respectively, while the prevalence of overweight risk was 12.1%, 5.2%, and 10.4%, respectively. Further details are provided in Table 1.

**Table 1:** Characteristics of Mothers and Their Children.

Characteristics	Participants, No. (%) [95%CI]
Mothers	
Maternal age, mean (SD) [range], y (*n* = 1,060)	30.1 (3.4) [20.0–49.0]
Mode of delivery (*n* = 1,149)	
Vaginal delivery	740 (64.4) [61.6–67.1]
Cesarean section	409 (35.6) [32.9–38.4]
Parity (*n* = 1,146)	
1	976 (85.2) [83.0–87.1]
2	170 (14.8) [12.9–17.0]
Children	
Birth weight, mean (SD) [range], g (*n* = 1,149)	3,366.7 (393.6) [1,970–4,850]
Low birth weight	10 (0.9) [0.5–1.6]
Macrosomia	64 (5.6) [4.4–7.1]
Breast-fed for 1 month (*n* = 1,146)	
Yes	953 (83.2) [80.9–85.2]
No	193 (16.8) [14.8–19.1]
Gestational age, mean (SD) [range], week	39.5 (1.0) [37–42]
BMI, mean (SD) [range]	
1 y	16.4 (1.3) [12.6–21.9]
2 y	15.1 (1.2) [10.9–22.5]
3 y	15.2 (1.4) [12.0–24.6]
Nutritional Status	
Wasting	
1 y	17 (1.5) [0.9–2.4]
2 y	77 (6.7) [5.4–8.3]
3 y	42 (3.7) [2.7–4.9]
Possible risk of overweight	
1 y	139 (12.1) [10.3–14.1]
2 y	60 (5.2) [4.1–6.7]
3 y	119 (10.4) [8.7–12.3]

BMI, Body Mass Index.

### 3.2 Comparison of birth weight classifications

As shown in Table 2, according to the CNGS, the proportions of infants classified as SGA, AGA, and LGA were 5.0% (*n* = 58), 81.4% (*n* = 935), and 13.6% (*n* = 156), respectively. In contrast, based on the IG-21 standards, the corresponding proportions were 3.0% (*n* = 35), 85.6% (*n* = 984), and 11.3% (*n* = 130). A total of 1,100 children (95.7%) were classified identically by both standards. Regarding the discrepancies, 23 children were identified as SGA by the CNGS but as AGA by the IG-21 standards, and 26 children were identified as LGA by the CNGS but as AGA by the IG-21 standards. The weighted kappa coefficient indicated strong agreement between the two standards, with a value of 0.856 (95% CI: 0.833–0.879; *P* < 0.001).

**Table 2:** Comparison of birth weight classifications between the CNGS and IG-21 standards.

CNGS	IG-21 standards			kappa	*P*
	SGA (35,3.0%)	AGA (984,85.6%)	LGA (130,11.3%)		
SGA (58,5.0%)	35	23	0	0.856 (0.833–0.879)	< 0.001
AGA (935,81.4%)	0	935	0		
LGA (156,13.6%)	0	26	130		

SGA, small for gestational age; AGA, appropriate for gestational age; LGA, large for gestational age; CNGS, Chinese newborn growth standards; IG-21 standards, International Fetal and Newborn Growth Consortium for the 21st Century standards.

**Figure 1:**
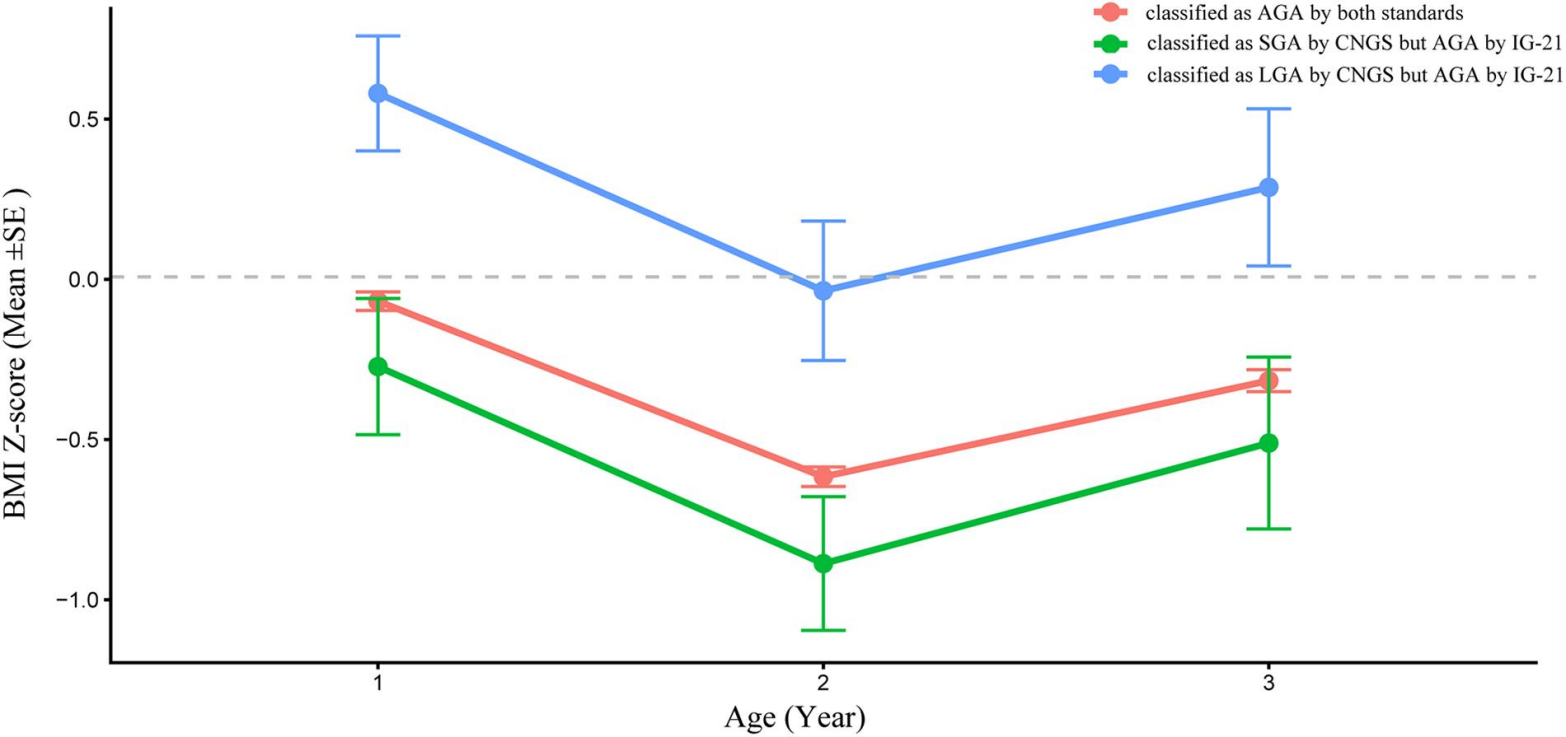
Comparison of BMI Z-score trajectories among children with different birth weight classifications.

Subgroup analyses were performed on children with discordant classifications between the two standards to compare their growth trajectories (Figure 1). Results showed that the BAZ of children classified as LGA by the CNGS but AGA by the IG-21 standards were significantly higher than those of the consistent AGA group at ages 1 and 2 years (*P* = 0.004 and *P* = 0.041, respectively), although the difference was not statistically significant at age 3 years (*P* = 0.066). In contrast, the BAZ of children classified as SGA by the CNGS but AGA by the IG-21 standards did not differ significantly from the consistent AGA group at any time point. This suggests that CNGS may be superior for identifying children at possible risk of overweight.

### 3.3 Offspring health outcomes

Table 3 presents the prevalence of wasting and possible risk of overweight stratified by birth weight categories. There were no statistically significant differences in the prevalence of these adverse nutritional outcomes between the groups defined by the CNGS and those defined by the IG-21 standards (all *P* > 0.05).

**Table 3:** Early childhood health outcomes under the CNGS and the IG-21 standards across different birth weight classifications.

Early childhood health outcomes	SGA		*P*	AGA		*P*	LGA		*P*
	CNGS (*n* = 58)	IG-21 standards (*n* = 35)		CNGS (*n* = 935)	IG-21 standards (*n* = 984)		CNGS (*n* = 156)	IG-21 standards (*n* = 130)	
Wasting									
1y	4 (6.9)	4 (11.4)	0.71	13 (1.4)	13 (1.3)	0.90	0 (0)	0 (0)	/
2y	12 (20.7)	9 (25.7)	0.57	64 (6.8)	67 (6.8)	0.98	1 (0.6)	1 (0.8)	1.00
3y	5 (8.6)	4 (11.4)	0.94	36 (3.9)	37 (3.8)	0.92	1 (0.6)	1 (0.8)	1.00
Possible risk of overweight									
1y	2 (3.4)	1 (2.9)	1.00	110 (11.8)	118 (12.0)	0.88	27 (17.3)	20 (15.4)	0.66
2y	1 (1.7)	0 (0)	1.00	43 (4.6)	49 (5.0)	0.70	16 (10.3)	11 (8.5)	0.61
3y	5 (8.6)	1 (2.9)	0.51	85 (9.1)	94 (9.6)	0.73	29 (18.6)	24 (18.5)	0.98

SGA, small for gestational age; AGA, appropriate for gestational age; LGA, large for gestational age; CNGS, Chinese newborn growth standards; IG-21 standards, International Fetal and Newborn Growth Consortium for the 21st Century standards.

### 3.4 Diagnostic utility of standards

Table 4 presents the association between birth weight classification (based on CNGS and IG-21 standards) and growth outcomes. Using modified Poisson regression models adjusted for potential confounders (including maternal age, parity, gestational complications, etc.), we calculated the RRs and 95% CIs for the wasting and possible risk of overweight in infants and toddlers classified as SGA or LGA, compared with the AGA group.

**Table 4:** RR and 95% CI of wasting and possible risk of overweight for SGA and LGA infants compared with the AGA group.

Outcome	SGA			Outcome	LGA		
	CNGS (*n* = 58)	IG-21 standards (*n* = 35)	*P*		CNGS (*n* = 156)	IG-21 standards (*n* = 130)	*P*
Wasting				Possible risk of overweight			
1y	4.975 (1.410–17.557)	8.235 (2.556–26.529)	< 0.001	1y	2.621 (0.998–6.879)	1.888 (0.638–5.590)	0.323
2y	3.601 (1.964–6.601)	4.290 (2.178–8.450)	0.137	2y	5.032 (1.446–17.506)	2.477 (0.625–9.816)	0.007
3y	2.452 (0.965–6.226)	3.419 (1.231–9.491)	0.170	3y	1.433 (0.588–3.491)	1.381 (0.544–3.505)	0.937

RR, relative risks; 95% CI, 95% confidence intervals; SGA, small for gestational age; AGA, appropriate for gestational age; LGA, large for gestational age; CNGS, Chinese newborn growth standards; IG-21 standards, International Fetal and Newborn Growth Consortium for the 21st Century standards.

*P*-values indicate the significance of the difference between the RRs estimated by the CNGS and IG-21 standards (test for heterogeneity).

Compared with AGA children, SGA defined by the CNGS was significantly associated with wasting at age 1 (RR, 4.975; 95% CI, 1.410–17.557) and age 2 (RR, 3.601; 95% CI, 1.964–6.601), but not at age 3. LGA defined by the CNGS was significantly associated with possible risk of overweight at age 2 (RR, 5.032; 95% CI, 1.446–17.506), with no significant associations at ages 1 or 3. Under the IG-21 standards, SGA was positively associated with wasting at all three ages, whereas LGA showed no significant association with possible risk of overweight at any age.

The 95% CIs for the RR calculated using the CNGS and IG-21 standards overlapped. Consequently, we conducted heterogeneity tests to statistically compare the RRs derived from the two standards. The results revealed significant differences in the prediction of wasting at 1 year and possible risk of overweight at 2 years, highlighting discrepancies between the two standards in evaluating nutritional status in infants and toddlers.

Table 5 presents the sensitivity, specificity, PPV, and NPV of the CNGS and IG-21 standards in predicting the nutritional status of infants and toddlers. Regarding the prediction of possible risk of overweight in children aged 1–3 years, the sensitivity of the CNGS was consistently higher than that of the IG-21 standards. This implies that LGA as defined by the Chinese standards is more effective in identifying children at possible risk of future overweight, thereby reducing missed diagnoses. This advantage was most pronounced at 2 years of age, where the sensitivity of the CNGS (45.5%) was 1.7 times that of the international standards (27.3%). Although the performance of both standards in predicting wasting was limited, Supplementary Table 2 indicates that the CNGS AGA classification demonstrated higher specificity for absence of overweight risk, suggesting that the Chinese standards are more effective in distinguishing children with normal growth trajectories from those at risk.

**Table 5:** Sensitivity, specificity, PPV, and NPV for SGA and LGA corresponding to each target event, under CNGS and IG-21 standards, respectively.

Target event and standards	Sensitivity (95%CI)	Specificity (95%CI)	PPV (95%CI)	NPV (95%CI)
Wasting				
1y				
IG-21 standards	0.235 (0.068–0.499)	0.973 (0.961–0.981)	0.114 (0.032–0.267)	0.988 (0.980–0.994)
CNGS	0.235 (0.068–0.499)	0.952 (0.938–0.964)	0.069 (0.019–0.167)	0.988 (0.980–0.994)
2y				
IG-21 standards	0.117 (0.055–0.210)	0.976 (0.965–0.984)	0.257 (0.125–0.433)	0.939 (0.923–0.952)
CNGS	0.156 (0.083–0.256)	0.957 (0.943–0.968)	0.207 (0.112–0.334)	0.940 (0.925–0.954)
3y				
IG-21 standards	0.095 (0.027–0.226)	0.972 (0.960–0.981)	0.114 (0.032–0.267)	0.966 (0.953–0.976)
CNGS	0.119 (0.040–0.256)	0.952 (0.938–0.964)	0.086 (0.029–0.190)	0.966 (0.954–0.976)
Possible risk of overweight				
1y				
IG-21 standards	0.235 (0.068–0.499)	0.889 (0.869–0.906)	0.031 (0.008–0.077)	0.987 (0.978–0.993)
CNGS	0.353 (0.142–0.617)	0.867 (0.846–0.887)	0.038 (0.014–0.082)	0.989 (0.980–0.994)
2y				
IG-21 standards	0.273 (0.060–0.610)	0.888 (0.869–0.906)	0.023 (0.005–0.066)	0.992 (0.985–0.997)
CNGS	0.455 (0.167–0.766)	0.867 (0.846–0.886)	0.032 (0.010–0.073)	0.994 (0.987–0.998)
3y				
IG-21 standards	0.167 (0.056–0.347)	0.888 (0.868–0.906)	0.038 (0.013–0.087)	0.975 (0.964–0.984)
CNGS	0.200 (0.077–0.386)	0.866 (0.845–0.885)	0.038 (0.014–0.082)	0.976 (0.964–0.984)

SGA, small for gestational age; AGA, appropriate-for-gestational-age; LGA, large-for-gestational-age; CNGS, Chinese newborn growth standards; IG-21 standards, International Fetal and Newborn Growth Consortium for the 21st Century standards; PPV, positive predictive value; NPV, negative predictive value.

## 4 Discussion

This study systematically compared the CNGS with the IG-21 standards regarding classification distribution and predictive validity for early childhood growth outcomes. Although the two standards demonstrated high overall agreement (weighted Kappa = 0.856), they exhibited clinically significant differences in the identification of high-risk infants. Specifically, LGA defined by the CNGS was significantly associated with possible risk of overweight at 2 years, an association not captured by the IG-21 standards. Furthermore, the CNGS exhibited higher sensitivity in identifying possible risk of overweight. These findings align with previous research emphasizing the importance of population-specific growth references (31), suggesting that international standards derived from diverse global populations may not offer optimal predictive accuracy for specific ethnic groups. This underscores the clinical value of localized standards.

The predictive utility of birth weight classifications is not static but is profoundly modulated by their interaction with underlying gestational risk factors. Beyond acting as a diagnostic marker, birth weight represents the culmination of the intrauterine environment, where specific pathologies dictate distinct postnatal growth trajectories. For instance, SGA infants born to mothers with advanced age or those experiencing threatened miscarriage often face a more compromised intrauterine milieu, which may exacerbate their vulnerability to persistent growth faltering or wasting (21, 25). Conversely, the interplay between LGA status and maternal gestational diabetes (GDM) is particularly critical; GDM-induced fetal hyperinsulinemia can trigger permanent shifts in metabolic programming, explaining why these LGA infants exhibit a significantly higher predisposition to childhood overweight compared to those born LGA due to other factors (27). Similarly, the developmental legacy of preeclampsia is complex: while it typically leads to lower initial weights, meta-analytic evidence reveals a paradoxical trend where these offspring are prone to rapid catch-up growth and increased adiposity as they mature (28). By integrating these prevalent epidemiological risk profiles—rather than relying on an “idealized” low-risk reference—the CNGS demonstrated superior sensitivity in identifying Chinese children whose early-life metabolic legacy places them on high-risk growth paths.

Our findings align closely with previous literature regarding the impact of birth weight on long-term health. Several studies have established that SGA is significantly associated with growth retardation in childhood (6, 32–34), while LGA significantly increases the risk of long-term obesity and related metabolic disorders (35, 36). Regarding physical development, research indicates that SGA children often face challenges in achieving full catch-up growth (37), with a reported risk of wasting was approximately 2-fold higher than that of AGA infants (38). Our study further quantified this risk, revealing a 4.98-fold and 3.60-fold increase in wasting at 1 and 2 years of age, respectively. Similarly, for LGA, a meta-analysis demonstrated a significantly elevated possible risk of overweight/obesity in early childhood (OR = 2.12; 95% CI: 1.22–3.70) (39). Consistent with these findings, our study confirmed that LGA children have a significantly higher possible risk of overweight, particularly at 2 years of age (RR = 5.032), further reinforcing the evidence for LGA as a robust predictor of long-term obesity.

Our findings are consistent with conclusions from similar studies suggesting that localized standards offer superior predictive utility. Research across various regions has demonstrated that birth weight standards derived from national population characteristics often outperform international standards in predicting local child health outcomes. For instance, a retrospective cohort study in Auckland, New Zealand, found that “customized birth weight standards” based on maternal characteristics were significantly superior to the IG-21 standards, which failed to identify a substantial number of at-risk SGA infants (40). In southern China, a localized head circumference reference was found to be more effective than the IG-21 standards in identifying neonates with microcephaly associated with genuine health risks (41). Similarly, a national study in Australia reported that birth weight references based on local multi-ethnic data were the most effective predictors of adverse perinatal outcomes (42). Collectively, evidence from these diverse settings suggests that while international standards provide a unified benchmark for global comparison, localized standards—which reflect the specific genetic background, environment, and nutritional status of a population—are of irreplaceable value for precision prevention and intervention in clinical practice.

### 4.1 Strengths and limitations

The major strengths of this study include the use of longitudinal follow-up data, which enabled a dynamic assessment of the association between birth weight categories and growth trajectories in infants and toddlers. Furthermore, we conducted a comprehensive comparison of the two standards across multiple dimensions, including agreement and predictive validity. These findings provide an empirical basis for the clinical utility of the CNGS.

However, this study also has limitations. First, participants from a single center may limit generalizability; larger, multi-center validation is needed. Second, our focus on anthropometric indicators warrants future inclusion of comprehensive outcomes like neurocognitive development and metabolic biomarkers. Third, as a retrospective study, unmeasured confounders (e.g., detailed socioeconomic status, dietary patterns) could not be fully adjusted. Fourth, while weight-for-length Z-score and age-adjusted BAZ showed good agreement, substituting the latter for children under two may introduce misclassification bias. This choice prioritized indicator uniformity in longitudinal analysis, and future research will consider refined body composition methods to address this limitation. Finally, our use of complete case analysis for missing covariate data, though straightforward, might introduce selection bias if not missing completely at random. Future studies will employ methods like multiple imputation and sensitivity analyses to ensure robustness.

## 5 Conclusion

In conclusion, although the CNGS and IG-21 standards showed high agreement in birth weight classification, they differed in their ability to predict growth outcomes in infants and toddlers. Notably, the CNGS demonstrated superior predictive validity, particularly higher sensitivity, in identifying possible risk of overweight in early childhood. Therefore, localized growth standards developed based on the specific characteristics of the population provide superior clinical utility for the early identification of growth deviations and the assessment of long-term health risks.

## Data Availability

The raw data supporting the conclusions of this article will be made available by the authors, without undue reservation.
