# Correction: Comparison of Chinese and international birth weight standards in predicting early childhood growth outcomes: a retrospective cohort study

**DOI:** 10.3389/fped.2026.1905752

**Published:** 2026-07-20

**Authors:** Ting Zhang, Zhenyu Zhang, Mai Gao, Ziyun Li, Li Zhang, Huijuan Liu, Yan Li

**Affiliations:** 1 Shandong Provincial Maternal and Child Health Care Hospital Affiliated to Qingdao University, Jinan, Shandong, China; 2 School of Clinical Medicine, Shandong Second Medical University, Weifang, Shandong, China

**Keywords:** birth weight, growth standards, possible risk of overweight, predictive validity, wasting

The “Equal Contribution Statement” for co-first authors **Ting Zhang and Zhenyu Zhang**, was erroneously omitted.

The original version of this article has been updated.

