# Supplementary material for: Comparison of Chinese and international birth weight standards in predicting early childhood growth outcomes: a retrospective cohort study
Source: Front Pediatr. 2026 Jun 8;14:1810686. doi: 10.3389/fped.2026.1810686 (PMC13284055; doi:10.3389/fped.2026.1810686)
Supplement: Supplementary file 1 [file Supplementaryfile1.docx]

| Supplementary Table 1. Agreement Between Weight-for-Length/Height Z-score and BAZ for Classifying Nutritional Status | | | | | | |
| --- | --- | --- | --- | --- | --- | --- |
|  |  | Weight-for length/height | | | Kappa | P |
|  |  | wasting | normal | possible risk of overweight |  |  |
| BAZ | wasting | 89 | 47 | 0 | 0.853 | <0.001 |
|  | normal | 5 | 2925 | 63 |  |  |
|  | possible risk of overweight | 0 | 6 | 312 |  |  |

Abbreviations: BMI-for-age Z-score.

| Supplementary Table 2. Sensitivity, Specificity, PPV, and NPV for AGA Corresponding to Each Target Event, Under CNGS and IG-21 standards, respectively | | | | |
| --- | --- | --- | --- | --- |
|  |  |  |  |  |
| Target event and standards | Sensitivity (95%CI) | Specificity (95%CI) | PPV (95%CI) | NPV (95%CI) |
| No Wasting |  |  |  |  |
| 1y |  |  |  |  |
| IG-21 standards | 0.858 (0.836–0.877) | 0.235 (0.096–0.473) | 0.987 (0.978–0.992) | 0.024 (0.009–0.061) |
| CNGS | 0.814 (0.791–0.836) | 0.235 (0.096–0.473) | 0.986 (0.976–0.992) | 0.019 (0.007–0.047) |
| 2y |  |  |  |  |
| IG-21 standards | 0.855 (0.833–0.875) | 0.130 (0.072–0.223) | 0.932 ( (0.914–0.946) | 0.061 (0.033–0.108) |
| CNGS | 0.812 (0.788–0.835) | 0.169 (0.101–0.268) | 0.932 ( (0.914–0.946) | 0.061 (0.036–0.101) |
| 3y |  |  |  |  |
| IG-21 standards | 0.855 (0.833–0.875) | 0.114 (0.050–0.240) | 0.960 (0.946–0.971) | 0.030 (0.013–0.069) |
| CNGS | 0.813 (0.789–0.835) | 0.159 (0.079–0.294) | 0.960 (0.946–0.971) | 0.033 (0.016–0.066) |
| No Risk of Overweight |  |  |  |  |
| 1y |  |  |  |  |
| IG-21 standards | 0.858 (0.836–0.877) | 0.235 (0.096–0.473) | 0.987 (0.978–0.992) | 0.024 (0.009–0.061) |
| CNGS | 0.817 (0.794–0.839) | 0.412 (0.216–0.640) | 0.989 (0.980–0.994) | 0.033 (0.016–0.066) |
| 2y |  |  |  |  |
| IG-21 standards | 0.858 (0.836–0.877) | 0.273 (0.097–0.566) | 0.992 (0.984–0.996) | 0.018 (0.006–0.052) |
| CNGS | 0.816 (0.793–0.838) | 0.455 (0.213–0.720) | 0.994 (0.986–0.997) | 0.023 (0.010–0.054) |
| 3y |  |  |  |  |
| IG-21 standards | 0.857 (0.835–0.876) | 0.161 (0.071–0.326) | 0.974 (0.962–0.982) | 0.030 (0.013–0.069) |
| CNGS | 0.815 (0.791–0.837) | 0.226 (0.114–0.398) | 0.974 (0.962–0.983) | 0.033 (0.016–0.066) |
| Abbreviations: SGA, Small for Gestational Age; AGA, Appropriate-for-Gestational-Age; LGA, Large-for-Gestational-Age; CNGS, Chinese Newborn Growth Standards; IG-21 standards, International Fetal and Newborn Growth Consortium for the 21st Century standards; PPV, positive predictive value; NPV, negative predictive value. | | | | |
